# Investigation on the Use and Preauthorization of Carbapenems at A National Hospital in Vietnam: A Cross‐Sectional Study

**DOI:** 10.1002/prp2.70145

**Published:** 2025-07-04

**Authors:** Han Ngoc Bao Pham, Nhi Truc Y. Nguyen, Nhu Quynh Tran, An Thi Thanh Nguyen, Cam Thi Le Pham, Nhan Thi Thanh Dang, Hien Thi Thu Pham, Thong Duy Vo, Mai Thi Phuong Tran, Quynh Thi Huong Bui

**Affiliations:** ^1^ Department of Clinical Pharmacy University of Medicine and Pharmacy Ho Chi Minh City Vietnam; ^2^ Department of Pharmacy Thong Nhat Hospital Ho Chi Minh City Vietnam; ^3^ Department of Internal Medicine, Faculty of Medicine University of Medicine and Pharmacy Ho Chi Minh City Vietnam; ^4^ Department of Gastroenterology University Medical Center Ho Chi Minh City Ho Chi Minh City Vietnam

**Keywords:** carbapenems, preauthorization, treatment outcome

## Abstract

Carbapenems, known for their broad‐spectrum activity and used as last‐line treatment for severe drug‐resistant infections, are encountering increasing resistance. Therefore, Thong Nhat Hospital, a national hospital in Vietnam, has implemented the preauthorization of some restricted antibiotics, including carbapenems, to optimize their use on patients. This study aimed to investigate the current use and preauthorization of carbapenems at the hospital. This descriptive cross‐sectional study was conducted on the medical records of inpatients having at least one order form for carbapenems from December 2023 to February 2024 at Thong Nhat Hospital. The data regarding microbiological features, carbapenem resistance patterns, characteristics of the use and preauthorization of carbapenems, and treatment outcome were collected for analysis. A total of 348 patients were selected for the study. The median age of patients was 69 years. *Escherichia coli* was the most frequently isolated Gram‐negative bacteria, and *Acinetobacter baumannii* had the lowest susceptibility to carbapenems. The appropriate rate of carbapenem regimens regarding the hospital guideline was 68.10%. Optimizing dosing was the most common intervention in preauthorization. The rate of patients whose preauthorization process of all restricted antimicrobials complied with the hospital's protocol was 73.28%. Factors associated with lower treatment success included high Charlson score and diagnoses of septic shock, respiratory tract infection, and central nervous system infection. The preauthorization of carbapenems should be enhanced to optimize the appropriate use of carbapenems, particularly in patients with multiple comorbidities and severe infections.

## 1 Introduction

In recent decades, the misuse and overuse of antibiotics have accelerated the emergence and spread of many drug‐resistant bacteria [1]. Among these antibiotics, carbapenems, known for their broad‐spectrum activity and used as a last‐line treatment for severe drug‐resistant infections, are encountering increasing resistance [2, 3]. Recognizing the urgency of this issue, the Infectious Diseases Society of America and the Society for Healthcare Epidemiology of America have introduced an effective measure—antimicrobial stewardship programs (ASP) to promote the appropriate use of antimicrobials and reduce resistance levels. One core strategy of ASP is preauthorization of restricted antimicrobials, requiring physicians to get approval for particular agents like carbapenems before prescribing them. This strategy has been proved to reduce the consumption of restricted antimicrobials and mitigate antibiotic resistance, particularly among Gram‐negative pathogens [4, 5]. Furthermore, carbapenems are classified in the WATCH group of the World Health Organization's AWaRE classification, underscoring the necessity of their judicious use and their priority as key targets for stewardship programs [6]. In light of these global initiatives, this study aimed to investigate the current use and preauthorization of carbapenems at a national hospital in Vietnam.

## 2 Materials and Methods

### 2.1 Study Design and Setting

A descriptive cross‐sectional study was conducted on the medical records of inpatients having at least one order form of carbapenems (ertapenem, imipenem/cilastatin, meropenem) from December 2023 to February 2024 at Thong Nhat Hospital, a national hospital in South Vietnam.

Inclusion criteria were as follows: (1) inpatients aged ≥ 18 years; (2) those with at least one order form of carbapenems (ertapenem, imipenem/cilastatin, meropenem, and doripenem) during the research period from December 2023 to February 2024. Patients who were pregnant or breastfeeding and those who absconded or transferred to another hospital for nonmedical reasons, were excluded. For patients with multiple hospital admissions during the study period, only the first admission's medical records were included.

The collected data included:

- Demographic characteristics: age, sex, body mass index (BMI), initial renal function (serum creatinine, creatinine clearance, and estimated glomerular filtration rate), comorbidities, infections, type of carbapenem regimen, and treatment outcome. BMI was defined as weight (kg) divided by the square of height (m) and was categorized into four groups according to the Asian‐Pacific cutoff points: underweight (< 18.5 kg/m^2^), normal weight (18.5–22.9 kg/m^2^), overweight (23.0–25.0 kg/m^2^), and obesity (≥ 25 kg/m^2^) [7]. Comorbidities are recorded based on diagnoses at hospital admission and medical history and evaluated using the Charlson Comorbidity Index [8]. Treatment outcome was defined as a condition at discharge classified into two groups: (1) Success: cured or improved, alleviated. (2) Failure: unchanged or worsened or deceased.
- Microbiological features: isolated pathogens and carbapenem susceptibility data.
- Characteristics of carbapenem regimens: appropriateness of indication, dosage, and route of administration.
- Characteristics of the restricted antimicrobials preauthorization: interventions and hospital's preauthorization protocol compliance.

### 2.2 Assessment of the Appropriateness of Carbapenem Regimens

We assessed the appropriateness of the first carbapenem regimen during the treatment period by examining the indication, dosage, and route of administration, based on the adherence to the hospital's guideline.

As for the indication, appropriateness was when the selection and combination of antimicrobials complied with the hospital's guidelines. Specifically, the selection of carbapenems in the empirical regimen was guided by the patient's risk for multidrug resistance. High‐risk patients received one of the following carbapenems: ertapenem, imipenem/cilastatin, meropenem, or doripenem. For moderate‐risk patients, ertapenem was preferred. However, other carbapenems could be used if there was a documented reason for the unavailability of first‐line empirical antibiotics or if consulted with the department head. For low‐risk patients, carbapenems could be considered only if the above criteria were met.

Dosage and administration criteria were only considered when the indication was appropriate.

The overall appropriateness of carbapenem regimens meant that patients received appropriate indications, dosages, and routes of administration.

### 2.3 Assessment of the Restricted Antimicrobials Preauthorization Compliance

The restricted antimicrobials preauthorization compliance was assessed at two levels.

- Individual order forms: compliance was determined if the preauthorization process of an order form followed the hospital's protocol. Noncompliance was considered if any step of this process was not followed the hospital's protocol.
- Patient cases: compliance was determined if all restricted antimicrobials used during treatment were preauthorized, and the preauthorization of all related order forms complied with the hospital's protocol. Other cases were considered noncompliant.

#### 2.3.1 The Hospital's Protocol of Restricted Antimicrobials' Preauthorization

The preauthorization is applied for agents classified in the hospital's restricted antimicrobials list and is conducted as follows: first, physicians are required to complete an order form before prescribing those antimicrobials, which is then sent to clinical pharmacists for online assessment. In cases of disagreement, the clinical pharmacist and physician will discuss to determine the most optimal regimen. Afterward, clinical pharmacists sign the final paper order form, and antimicrobials are dispensed.

All order forms must be assessed by clinical pharmacists before patients' administration. In emergencies or outside regular working hours (e.g., overnight shifts and weekends), the antimicrobials can be dispensed with approval from the clinical department head. These forms must still be assessed by clinical pharmacists on the next working day.

### 2.4 Statistical Analysis

- Continuous variables with normal distributions were presented as mean ± standard deviation, while non‐normal distributions were presented as median (interquartile range).
- Nominal variables were described by frequency and percentage.
- Multivariate logistic regression with the backward conditional method was used to assess factors related to treatment outcome. The dependent variable was success or failure. Independent variables, including age, sex, BMI, estimated glomerular filtration rate, number and types of infections, Charlson score, appropriateness of carbapenem regimens, and hospital's preauthorization protocol compliance, were examined for correlation using Pearson's or Spearman's test. Variables with *r* < 0.6 were included in the regression model, while for *r* ≥ 0.6, only one variable was selected. Data were analyzed by IBM SPSS Statistics version 26 and Microsoft Excel 2016. *p*‐values < 0.05 were considered statistically significant.

### 2.5 Ethics Approval

The protocol of this study was approved by the Institutional Review Board of Thong Nhat Hospital, Ho Chi Minh City, Vietnam (129/2023/BVTN‐HDDD).

## 3 Results

### 3.1 Demographic Characteristics

Of the 421 patients meeting the inclusion criteria, 348 were included in the study after applying the exclusion criteria. The majority of patients were elderly (≥ 60 years), with a median age of 69 (52–82). In total, nearly 60% of patients were men. Patients recorded eGFR ≤ 60 mL/min/1.73 m^2^ at the time of admission accounted for approximately 40%. The median number of comorbidities was 3 (2–5) and the Charlson Comorbidity Index score was 1 (0–2). The most common comorbidities were hypertension (62.64%) and diabetes mellitus (39.37%). Sepsis and septic shock are among the most prevalent infections encountered during treatment (47.41%) (Table 1).

**TABLE 1:** Patients' demographic characteristics (*n* = 348).

Characteristics	Median (IQR) or *n* (%) of patients
Age (years)	69 (52–82)
< 60	111 (31.90)
≥ 60	237 (68.10)
Sex	
Male	207 (59.48)
Female	141 (40.52)
Body mass index (kg/m^2^) (*n* = 312)	22.2 (19.7–24.7)
Underweight	38 (12.18)
Normal weight	141 (45.19)
Overweight	59 (18.91)
Obesity	74 (23.72)
Initial renal function	
SCr (μmol/L)	92.00 (75.00–128.5)
CrCl (mL/min)	41.00 (30.40–66.95)
eGFR (mL/min/1.73 m^2^)	66.99 (48.49–86.62)
≤ 60	142 (40.80)
> 60	206 (59.20)
Number of comorbidities	3 (2–5)
Charlson score	1 (0–2)
Types of comorbidities	
Cardiovascular disease	228 (65.52)
Hypertension	218 (62.64)
Other cardiovascular diseases	126 (36.21)
Diabetes mellitus	137 (39.37)
Hyperlipidemia	83 (23.85)
Gastroesophageal reflux disease	70 (20.11)
Chronic kidney disease	51 (14.66)
Poststroke sequelae	43 (12.36)
Cancer	40 (11.49)
Liver disease	26 (7.47)
Chronic obstructive pulmonary disease	12 (3.45)
Asthma	9 (2.59)
Others	214 (61.49)
Number of infections	1 (1–2)
Type of infections	
Sepsis/septic shock	165 (47.41)
Respiratory tract infection	152 (43.68)
Community‐acquired pneumonia	101 (29.02)
Hospital‐acquired pneumonia	47 (13.51)
Ventilator‐associated pneumonia	4 (1.15)
Urinary tract infection	114 (32.76)
Intra‐abdominal infection	86 (24.71)
Skin and soft tissue infection	53 (15.22)
Central nervous system infection	14 (4.02)
Infective endocarditis	1 (0.28)
Type of carbapenem regimen	
Empirical	193 (55.46)
Alternative	155 (44.54)
Treatment outcome	
Success	293 (84.20)
Failure	55 (15.80)

Abbreviations: CrCl, creatinine clearance; GFR, estimated glomerular filtration rate; IQR, interquartile range; SCr, serum creatinine.

### 3.2 Microbiological Features

Among 348 patients, 303 had samples collected for microbiological testing during treatment, of which 76.57% had samples taken before the administration of empirical antibiotics. Of the 294 pathogens isolated from 303 patients, the majority were Gram‐negative bacteria (68.37%), with *E. coli* being the most prevalent (Table 2).

**TABLE 2:** Isolated pathogens isolated (*n* = 294).

Organism	Number of isolates, *n* (%)
Gram‐negative bacteria	201 (68.37)
*Escherichia coli*	77 (26.19)
*Klebsiella pneumoniae*	47 (15.99)
*Pseudomonas aeruginosa*	21 (7.14)
*Acinetobacter baumannii*	20 (6.80)
*Enterobacter cloacae*	8 (2.72)
*Klebsiella aerogenes*	7 (2.38)
*Stenotrophomonas maltophilia*	4 (1.36)
*Achromobacter denitrificans*	3 (1.02)
*Proteus mirabilis*	3 (1.02)
*Achromobacter xylosoxidans*	2 (0.68)
*Burkholderia pseudomallei*	2 (0.68)
*Klebsiella ozaenae*	2 (0.68)
*Morganella morganii*	2 (0.68)
*Salmonella* spp.	2 (0.68)
*Acinetobacter junii*	1 (0.34)
Gram‐positive bacteria	61 (20.75)
*Staphylococcus aureus*	37 (12.59)
*Staphylococcus epidermidis*	6 (2.04)
*Staphylococcus haemolyticus*	5 (1.70)
*Enterococcus faecium*	5 (1.70)
*Staphylococcus hominis*	4 (1.36)
*Streptococcus sanguinis*	2 (0.68)
*Staphylococcus warneri*	1 (0.34)
*Enterococcus faecalis*	1 (0.34)
Fungi	32 (10.88)
*Candida albicans*	17 (5.78)
*Candida tropicalis*	12 (4.08)
*Candida guilliermondii*	2 (0.68)
*Candida krusei*	1 (0.34)

Most Gram‐negative bacteria isolated demonstrated high susceptibility (≥ 80%) to ertapenem (Table 3). Among four commonly isolated Gram‐negative strains, *E. coli* was notably sensitive to all three carbapenems, including ertapenem, imipenem, and meropenem. In contrast, *Klebsiella pneumoniae* and *Pseudomonas aeruginosa* showed lower susceptibility to these antibiotics. *Acinetobacter baumannii* had the lowest susceptibility to carbapenems (< 10%).

**TABLE 3:** Carbapenem susceptibility of common Gram‐negative bacteria.

Susceptibility rate (%)	Ertapenem	Imipenem	Meropenem
*Escherichia coli*	96.88	90.79	96.55
*Klebsiella pneumoniae*	80.00	57.78	44.00
*Pseudomonas aeruginosa*	—	52.38	52.38
*Acinetobacter baumannii*	—	7.14	6.67
Other Enterobacterales^a^	87.50	36.84	75.00

^a^ Other Enterobacterales: *Enterobacter cloacae* , Klebsiella aerogenes, *Klebsiella ozaenae* , *Morganella morganii* , *Proteus mirabilis* , *Salmonella* spp.

### 3.3 Assessment of the Appropriateness of Carbapenem Regimens

According to the hospital's guidelines, carbapenem regimens were appropriate in 237 (68.10%) patients. Specifically, the alternative regimens demonstrated higher adherence to prescribing criteria, including indication and dosage, compared to empirical ones (Table 4).

**TABLE 4:** Appropriateness of carbapenem regimens (*n* = 348).

Criteria, *n* (%)	Total cases (*n* = 348)	Empirical regimens (*n* = 193)	Alternative regimens (*n* = 155)
Indication	320 (91.95)	176 (91.19)	144 (92.90)
Dosage^a^	239 (74.69)	127 (72.17)	112 (77.78)
Route of administration^a^	319 (99.69)	176 (100.00)	143 (99.31)
Overall	237 (68.10)	126 (65.28)	111 (71.61)

^a^ Dosage and administration criteria were only considered when the indication was appropriate.

In 11 cases where the selection of carbapenems for empirical regimens was deemed inappropriate, group 2 carbapenems, specifically imipenem/cilastatin and meropenem, were prescribed for patients at low to moderate risk of multidrug‐resistant bacterial infections, while the recorded data did not identify any clinical factors warranting the use of carbapenems as first‐line empirical therapy. Besides, the indication for alternative regimens often lacked proper justification, accounting for more than half of cases, compared to only 17.65% in empirical regimens (Table 5).

**TABLE 5:** Reasons for inappropriateness of carbapenem regimens.

Criteria, *n* (%)	Empirical regimens	Alternative regimens
Indication	***n* = 17**	***n* = 11**
No indication for use	3 (17.65)	6 (54.55)
Inappropriate type of carbapenem selection	11 (64.70)	—
Redundant antibiotic combinations	3 (17.65)	4 (36.36)
Deficient antibiotic combinations	0 (0.00)	1 (9.09)
Dosage	***n* = 49**	***n* = 32**
Carbapenem	19 (38.78)	11 (34.38)
Combined agents	18 (36.73)	13 (40.63)
Both	12 (24.49)	8 (25.00)
Route of administration	***n* = 0**	***n* = 1**
Combined agents	—	1 (100.00)

### 3.4 The Restricted Antimicrobials Preauthorization

Throughout the treatment of 348 patients, a total of 473 order forms of restricted antimicrobials were collected. Among these forms, 395 included carbapenems, while 78 involved other restricted antibiotics without carbapenems. A total of 129 interventions were made. Optimizing dosing was the main intervention (83.72%), followed by a change to another antibiotic (10.85%), exclusion of unnecessary antibiotics (3.10%), and antibiotic addition (2.33%). Intervention acceptance rates were low, at 59.26%, 14.29%, 50.00%, and 33.33%, respectively. The interventions recommended and rates of acceptance were described in Table 6.

**TABLE 6:** Interventions and acceptance rates (*n* = 129).

Criteria, *n* (%)	Total	Carbapenem order forms	Other restricted antimicrobials order forms
	*n* = 129	*n* = 111	*n* = 18
Interventions recommended			
Optimize dosing	108 (83.72)	93 (83.79)	15 (83.33)
Change antibiotic	14 (10.85)	12 (10.81)	2 (11.11)
Exclude unnecessary antibiotic	4 (3.10)	3 (2.70)	1 (5.56)
Add antibiotic	3 (2.33)	3 (2.70)	0 (0.00)
Interventions accepted			
Optimize dosing	64 (59.26)	56 (60.22)	8 (53.33)
Change antibiotic	2 (14.29)	2 (16.67)	0 (0.00)
Exclude unnecessary antibiotic	2 (50.00)	2 (66.67)	0 (0.00)
Add antibiotic	1 (33.33)	1 (33.33)	—

The main reason order forms were considered noncompliant was due to delays in submitting order forms in cases that were not emergencies or outside regular working hours (Table 7).

**TABLE 7:** The compliance of restricted antimicrobials preauthorization.

	*n* (%)
Individual order forms (*n* = 473)	
Compliance	377 (79.70)
Noncompliance	96 (20.30)
Not send order forms online	38 (8.04)
Send order forms after administration	58 (12.26)
Patient cases (*n* = 348)	
Compliance	255 (73.28)
Noncompliance	93 (26.72)

### 3.5 Logistic Regression Analysis

Based on the correlation analysis of independent variables, the following variables were included in the multivariate regression model: age, sex, eGFR, Charlson score, sepsis, septic shock, respiratory tract infection, central nervous system infection, urinary tract infection, intra‐abdominal infection, skin and soft tissue infection, appropriateness of carbapenem regimens, and compliance with the hospital's preauthorization protocol.

Multivariable logistic regression using the backward method was performed in Table 8. High Charlson score and diagnoses of septic shock, respiratory tract infection, and central nervous system infection were independent predictors of a lower treatment success rate.

**TABLE 8:** Factors related to the treatment outcome (*n* = 348).

Factor		OR	95% CI		*p*
			Upper	Lower	
eGFR		0.992	0.982	1002	0.107
Charlson score		0.819	0.705	0.952	**0.009**
Septic shock	Yes	0.093	0.029	0.301	**< 0.001**
Sepsis	Yes	0.508	0.252	1022	0.057
Respiratory tract infection	Yes	0.237	0.115	0.486	**< 0.001**
Central nervous system infection	Yes	0.161	0.043	0.594	**0.006**

*Note:* Bold values indicate statistically significant results.

Abbreviations: CI, confidence interval; eGFR, estimated glomerular filtration rate; OR, odds ratio.

## 4 Discussion

The patients in our study had a relatively high age range, with a median age of 69 (52–82). This can be attributed to our hospital's specialization in geriatric care, leading to a predominance of elderly patients. Carbapenems, preferred for treating severe infections or infections caused by drug‐resistant bacteria, are commonly used in this population, who are at higher risk of drug‐resistant bacterial infections due to immunosenescence and multiple comorbidities [9]. Approximately 40.80% of the study population presented with renal impairment, as indicated by an estimated glomerular filtration rate of less than 60 mL/min/1.73 m^2^. Renal dysfunction not only compromises the body's immune defenses, thereby worsening infection outcomes, but also significantly impacts the pharmacokinetics and pharmacodynamics of antibiotics [10, 11]. Given that carbapenems are predominantly renally excreted, it is essential to adjust dosing based on creatinine clearance. Failure to appropriately modify doses in accordance with renal function may elevate the risk of adverse effects, including nephrotoxicity and seizures [12, 13].

### 4.1 Microbiological Features

Our findings reveal a remarkable disparity in carbapenem susceptibility profiles among the isolated pathogens. While *E. coli* demonstrated relatively high susceptibility rates, *K. pneumoniae* , *P. aeruginosa* , and *A. baumannii* exhibited significantly lower susceptibility. These results are consistent with the findings of Aloraifi et al. [14], showing the highest rates of carbapenem resistance in *A. baumannii* and the lowest in *E. coli* . The similarities between our study and the previous research underscore the alarming prevalence of carbapenem resistance in *A. baumannii* . Antimicrobial resistance is a critical determinant of treatment outcome in infections caused by *A. baumannii* [15]. Therefore, the implementation of ASP, including the preauthorization of restricted antimicrobials, is imperative. Such interventions have been shown to effectively delay the emergence of multidrug‐resistant *A. baumannii* and improve treatment success rates [16].

### 4.2 Assessment of the Appropriateness of Carbapenem Regimens

In empirical regimens, the appropriateness of indication and route of administration was high, at 91.19% and 100%, respectively. Inappropriate indications mainly resulted from the incorrect selection of carbapenems, not aligned with the patient's risk of multidrug‐resistant bacterial infections. The prescription of carbapenems should be carefully considered based on the risk of resistant bacteria to avoid selecting resistant strains or insufficient coverage, such as using ertapenem in cases with risk factors for *Pseudomonas* or other nonfermenting bacteria [17].

The appropriateness of the alternative regimens (71.61%) was higher than the empirical regimens (65.28%). This is likely because carbapenems in the alternative regimens were used for patients with worsening infections or prior antibiotic failure. As a result, the escalation to carbapenem therapy was likely considered more carefully in these patients.

### 4.3 The Restricted Antimicrobials Preauthorization

During the treatment period, 73.28% of patients and 79.70% of order forms complied with the hospital's restricted antimicrobials preauthorization, reflecting strong physician adherence to this process. Close collaboration between physicians and clinical pharmacists in prescribing antibiotics helps optimize their safe, effective, and guideline‐based use.

In our study, most interventions focused on optimizing dosing, while a study by Seah et al. [18] found that the most common intervention was carbapenem discontinuation (35%), while optimization of dosing accounted for only 17%. This discrepancy may be explained by the reasons for inappropriate carbapenem use in Seah's study, such as prolonged treatment duration or unclear indications, whereas our study had a higher rate of appropriate indications (> 90%). We also found that optimizing dosing was the most accepted intervention by physicians. In contrast, interventions such as discontinuing carbapenems or switching to narrower‐spectrum antibiotics were often rejected. Similar results were observed in a previous study by Seah et al. [18] (65.40% compared to 35.10% and 40%). This reluctance may be attributed to physicians' prescribing experience. Carbapenems, with their broad‐spectrum activity, are effective against a wide range of infections, including multidrug‐resistant bacteria [19]. As a result, they are frequently used as an escalation approach, particularly when clinical improvement or the risk of drug resistance is unclear. However, overuse of broad‐spectrum antibiotics like carbapenems can contribute to the selection of resistant bacterial strains, potentially complicating future treatment options [20].

### 4.4 Logistic Regression Analysis

We found that only four factors were significantly associated with treatment outcome in the multiple regression analysis (*p* < 0.05). First, a higher Charlson score was a significant predictor of poor treatment outcome. The Charlson Comorbidity Index includes 19 conditions like diabetes, kidney failure, COPD, and cancer, which are key risk factors for multidrug‐resistant bacterial infections, complicating the treatment.

Second, patients diagnosed with septic shock are associated with lower treatment success rates. This result can be explained by the fact that septic shock, a severe form of sepsis involving circulatory and cellular‐metabolic dysfunction, poses a significantly higher risk of mortality [21]. A systematic review and meta‐analysis by Bauer et al. [22] in Europe, North America, and Australia reported that the mortality rate due to septic shock within 30 days was 34.73% and within 90 days was 38.47%.

Third, respiratory tract infection was a factor associated with treatment failure. Similarly, in a study by Rattanaumpawan et al. [23], the multivariate analysis demonstrated a significant association between respiratory tract infection and unfavorable clinical outcomes (OR: 2.36, 95% CI: 1.78–3.15, *p* < 0.001).

Finally, central nervous system infection was significantly associated with reduced treatment success rates. Previous studies have reported that bacterial meningitis is among the leading causes of infection‐related mortality. Mortality rates for meningitis can reach up to 30%, with acute pneumococcal meningitis showing a mortality range of 6%–17%. A retrospective study by Niemelä et al. in Finland reported a 40.5% treatment failure rate in meningitis patients, with a 30‐day mortality rate of 10.8% [24, 25].

## 5 Conclusion

The preauthorization of carbapenems should be enhanced to optimize the appropriate use of carbapenems, particularly in patients with multiple comorbidities and severe infections.

## Author Contributions

H.N.B.P., N.T.Y.N., N.Q.T., A.T.T.N., C.T.L.P., N.T.T.D., H.T.T.P., T.D.V., M.T.P.T., and Q.T.H.B. contributed to the study conception, design, data collection, analysis, and manuscript writing. H.N.B.P., T.D.V., and Q.T.H.B. revised the manuscript. Q.T.H.B. supervised the study and critically revised the manuscript. All authors reviewed and approved the final version of the manuscript.

## Ethics Statement

The protocol of this study was approved by the Institutional Review Board of Thong Nhat Hospital, Ho Chi Minh City, Vietnam on November 30, 2023, No. 129/2023/BVTN‐HDDD.

## Conflicts of Interest

The authors declare no conflicts of interest.

## Data Availability

Upon reasonable request, the datasets of this study can be made available from the corresponding author.

## References

[1] “Antimicrobial Stewardship Programmes in Health‐Care Facilities in Low‐ and Middle‐Income Countries: A WHO Practical Toolkit,” JAC‐Antimicrobial Resistance 1, no. 3 (2019): dlz072. 34222945 10.1093/jacamr/dlz072 PMC8210188

[2] T. Armstrong , S. J. Fenn , and K. R. Hardie , “JMM Profile: Carbapenems: A Broad‐Spectrum Antibiotic,” Journal of Medical Microbiology 70, no. 12 (2021): 001462. 34889726 10.1099/jmm.0.001462 PMC8744278

[3] F. S. Codjoe and E. S. Donkor , “Carbapenem Resistance: A Review,” Medical Sciences (Basel, Switzerland) 6, no. 1 (2017): 1. 29267233 10.3390/medsci6010001 PMC5872158

[4] T. H. Dellit , R. C. Owens , J. E. McGowan, Jr. , et al., “Infectious Diseases Society of America and the Society for Healthcare Epidemiology of America Guidelines for Developing an Institutional Program to Enhance Antimicrobial Stewardship,” Clinical Infectious Diseases: An Official Publication of the Infectious Diseases Society of America 44, no. 2 (2007): 159–177. 17173212 10.1086/510393

[5] T. F. Barlam , S. E. Cosgrove , L. M. Abbo , et al., “Implementing an Antibiotic Stewardship Program: Guidelines by the Infectious Diseases Society of America and the Society for Healthcare Epidemiology of America,” Clinical Infectious Diseases 62, no. 10 (2016): e51–e77. 27080992 10.1093/cid/ciw118 PMC5006285

[6] World Health Organization , “AWaRe Classification of Antibiotics for Evaluation and Monitoring of Use, 2023,” 2023, https://www.who.int/publications/i/item/WHO‐MHP‐HPS‐EML‐2023.04.

[7] W. H. Pan and W. T. Yeh , “How to Define Obesity? Evidence‐Based Multiple Action Points for Public Awareness, Screening, and Treatment: An Extension of Asian‐Pacific Recommendations,” Asia Pacific Journal of Clinical Nutrition 17, no. 3 (2008): 370–374. 18818155

[8] C. E. Roffman , J. Buchanan , and G. T. Allison , “Charlson Comorbidities Index,” Journal of Physiotherapy 62, no. 3 (2016): 171. 27298055 10.1016/j.jphys.2016.05.008

[9] H. J. Hepper , C. Sieber , P. Walger , P. Bahrmann , and K. Singler , “Infections in the Elderly,” Critical Care Clinics 29, no. 3 (2013): 757–774. 23830661 10.1016/j.ccc.2013.03.016

[10] M. Syed‐Ahmed and M. Narayanan , “Immune Dysfunction and Risk of Infection in Chronic Kidney Disease,” Advances in Chronic Kidney Disease 26, no. 1 (2019): 8–15. 30876622 10.1053/j.ackd.2019.01.004

[11] F. Keller and A. Hann , “Clinical Pharmacodynamics: Principles of Drug Response and Alterations in Kidney Disease,” Clinical Journal of the American Society of Nephrology: CJASN 13, no. 9 (2018): 1413–1420. 29769182 10.2215/CJN.10960917 PMC6140566

[12] S. R. Norrby , “Neurotoxicity of Carbapenem Antibacterials,” Drug Safety 15, no. 2 (1996): 87–90. 8884160 10.2165/00002018-199615020-00001

[13] D. P. Nicolau , “Carbapenems: A Potent Class of Antibiotics,” Expert Opinion on Pharmacotherapy 9, no. 1 (2008): 23–37. 18076336 10.1517/14656566.9.1.23

[14] R. I. Aloraifi , A. F. Alharthi , A. A. Almefleh , et al., “Prevalence of Carbapenem Non‐Susceptible Gram‐Negative Bacteria at Tertiary Care Hospitals in Saudi Arabia,” Cureus 15, no. 1 (2023): e33767. 36655153 10.7759/cureus.33767 PMC9840728

[15] H. Y. Lee , C. L. Chen , S. R. Wu , C. W. Huang , and C. H. Chiu , “Risk Factors and Outcome Analysis of *Acinetobacter baumannii* Complex Bacteremia in Critical Patients,” Critical Care Medicine 42, no. 5 (2014): 1081–1088. 24394630 10.1097/CCM.0000000000000125

[16] M. Nguyen and S. G. Joshi , “Carbapenem Resistance in *Acinetobacter baumannii*, and Their Importance in Hospital‐Acquired Infections: A Scientific Review,” Journal of Applied Microbiology 131, no. 6 (2021): 2715–2738. 33971055 10.1111/jam.15130

[17] P. M. Hawkey and D. M. Livermore , “Carbapenem Antibiotics for Serious Infections,” BMJ (Clinical Research Ed) 344 (2012): e3236. 10.1136/bmj.e3236 22654063

[18] V. X. F. Seah , R. Y. L. Ong , A. S. Y. Lim , C. Y. Chong , N. W. H. Tan , and K. C. Thoon , “Impact of a Carbapenem Antimicrobial Stewardship Program on Patient Outcomes,” Antimicrobial Agents and Chemotherapy 61, no. 9 (2017): e00736‐17. 28717037 10.1128/AAC.00736-17 PMC5571328

[19] M. I. El‐Gamal , I. Brahim , N. Hisham , R. Aladdin , H. Mohammed , and A. Bahaaeldin , “Recent Updates of Carbapenem Antibiotics,” European Journal of Medicinal Chemistry 131 (2017): 185–195. 28324783 10.1016/j.ejmech.2017.03.022

[20] A. F. Alsehemi , E. A. Alharbi , B. B. Alammash , A. I. Alrais , H. M. Elbadawy , and Y. M. Alahmadi , “Assessment of Risk Factors Associated With Multidrug‐Resistant Organism Infections Among Patients Admitted in a Tertiary Hospital – A Retrospective Study,” Saudi Pharmaceutical Journal: SPJ: The Official Publication of the Saudi Pharmaceutical Society 31, no. 6 (2023): 1084–1093. 37293381 10.1016/j.jsps.2023.03.019 PMC10244471

[21] R. S. Hotchkiss , L. L. Moldawer , S. M. Opal , K. Reinhart , I. R. Turnbull , and J. L. Vincent , “Sepsis and Septic Shock,” Nature Reviews Disease Primers 2 (2016): 16045. 10.1038/nrdp.2016.45 PMC5538252 28117397

[22] M. Bauer , H. Gerlach , T. Vogelmann , F. Preissing , J. Stiefel , and D. Adam , “Mortality in Sepsis and Septic Shock in Europe, North America and Australia Between 2009 and 2019 – Results From a Systematic Review and Meta‐Analysis,” Critical Care (London, England) 24, no. 1 (2020): 239. 32430052 10.1186/s13054-020-02950-2 PMC7236499

[23] P. Rattanaumpawan , P. Sutha , and V. Thamlikitkul , “Effectiveness of Drug Use Evaluation and Antibiotic Authorization on Patients' Clinical outcomes, Antibiotic Consumption, and Antibiotic Expenditures,” American Journal of Infection Control 38, no. 1 (2010): 38–43. 19699014 10.1016/j.ajic.2009.04.288

[24] E. K. Gudina , M. Tesfaye , A. Wieser , H. W. Pfister , and M. Klein , “Outcome of Patients With Acute Bacterial Meningitis in a Teaching Hospital in Ethiopia: A Prospective Study,” PLoS One 13, no. 7 (2018): e0200067. 30020952 10.1371/journal.pone.0200067 PMC6051621

[25] S. Niemelä , L. Lempinen , E. Löyttyniemi , J. Oksi , and J. Jero , “Bacterial Meningitis in Adults: A Retrospective Study Among 148 Patients in an 8‐Year Period in a University Hospital, Finland,” BMC Infectious Diseases 23, no. 1 (2023): 45. 36690945 10.1186/s12879-023-07999-2 PMC9869503

